# Osteoporosis in Iraqi patients with thalassemia

**DOI:** 10.1186/ar3605

**Published:** 2012-02-09

**Authors:** Salim M AL Jadir, Mohamed Z Jalal, Median F AL Ghreer, Mozahem S AL Hamdani, Wamid R AL Omaree

**Affiliations:** 1 Al Salaam General Teaching Hospital in Mosul City, Iraq; 2 Ibn Al Atheer Teaching Hospital, Thalassemia center, Mosul City, Iraq; 3 Medical College of Mosul UNV, Mosul, Iraq

## Background

Thalassemia is defined as a complete absence of one or more of the four globins in the red blood cells due to the deletion of or nonfunctioning of one or more genes.

Osteoporosis is a universal medical problem, affecting both genders.

## Materials and methods

74 thalassemic patients 36 male and 38 female below the age of 25 years.

The study was a clinical cross-sectional for both genders with thalassemia major, Investigation done included a chest × ray, serum iron, total iron binding capacity (TIBC), transferrin saturation, serum calcium, serum phosphorus, serum alkaline phosphatase, blood urea, serum creatinine, and a DXA bone scan.

Statistical analysis:-P-value--S.P.S.S.--chi-square.

## Results

We found that the bony disorder in thalassemic patients increased with age (bone pain, carpopedal spasm, osteoporosis), and with low serum iron and low T.I.B.C. and with increased transferrin saturation. The compliance of patients with treatment was rated as in 24 good, in 36 fair and in 14 bad.

The prevalence of osteoporosis in thalassemic Iraqi patients DXA scans was found to be 67.5% while osteopenia was found in 9.4% and normal BMD in 22.9%.

## Discussion

During the last decade, the presence of osteopenia and osteoporosis in well-treated thalassaemics has been described in different studies with high prevalence up to 50%.

Several factors are implicated in reduction of bone mass in thalassaemia major. Delayed sexual maturation, growth hormone (GH) and insulin growth factor-(IGF)-1 deficiency, parathyroid gland dysfunction, diabetes, hypothyroidism, ineffective haemopoiesis with progressive marrow expansion, direct iron toxicity on osteoblasts, as well as liver disease have been indicated as possible etiological factors for thalassaemia-induced osteoporosis. Furthermore, iron chelating has correlated with growth failure and bone abnormalities, and high desferrioxamine dosage has been associated with cartilage alterations.

## Conclusions

Osteoporosis in thalassemic Iraqi patient was too high and even more in those patients with bad compliance regard attendance to the Thalassemia centre.

## Recommendations

We need to inform the thalassemic patients about the risk of osteoporosis and the need for their awareness regard such complication and the importance of their compliance with therapy.

